# The Arctic-Subarctic sea ice system is entering a seasonal regime: Implications for future Arctic amplification

**DOI:** 10.1038/s41598-017-04573-0

**Published:** 2017-07-04

**Authors:** Thomas W. N. Haine, Torge Martin

**Affiliations:** 1 Earth & Planetary Sciences, The Johns Hopkins University, Baltimore, MD USA; 2 GEOMAR Helmholtz Centre for Ocean Research Kiel, Kiel, Germany

## Abstract

The loss of Arctic sea ice is a conspicuous example of climate change. Climate models project ice-free conditions during summer this century under realistic emission scenarios, reflecting the increase in seasonality in ice cover. To quantify the increased seasonality in the Arctic-Subarctic sea ice system, we define a non-dimensional seasonality number for sea ice extent, area, and volume from satellite data and realistic coupled climate models. We show that the Arctic-Subarctic, i.e. the northern hemisphere, sea ice now exhibits similar levels of seasonality to the Antarctic, which is in a seasonal regime without significant change since satellite observations began in 1979. Realistic climate models suggest that this transition to the seasonal regime is being accompanied by a maximum in Arctic amplification, which is the faster warming of Arctic latitudes compared to the global mean, in the 2010s. The strong link points to a peak in sea-ice-related feedbacks that occurs long before the Arctic becomes ice-free in summer.

## Introduction

**Figure 1:**
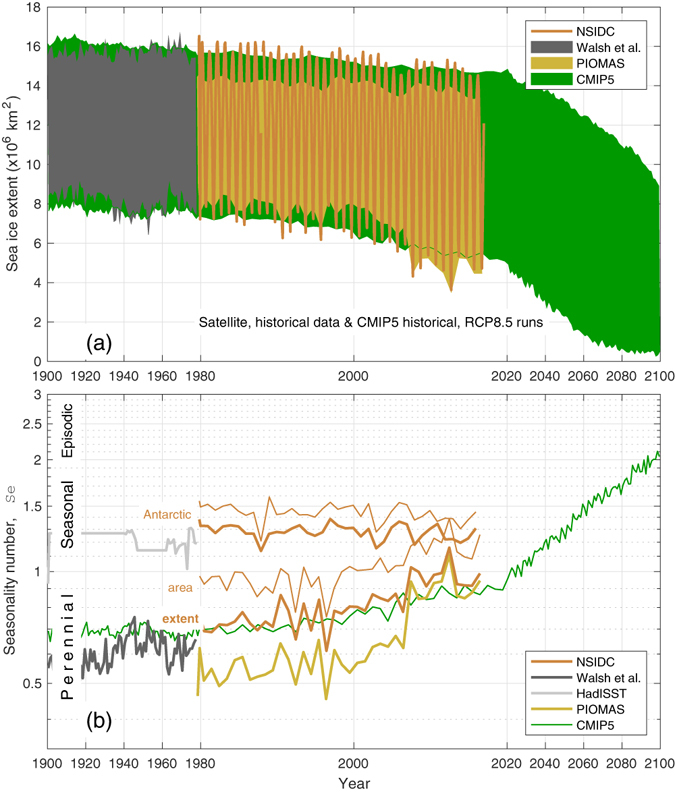
Increasing seasonality of Arctic sea ice extent. (**a**) Annual range of sea ice extent from data and model simulations, (**b**) The corresponding seasonality numbers. Monthly NSIDC satellite sea ice index data are shown since 1978 and the Walsh *et al*.^19^ composite of historical data are shown prior to 1978. The PIOMAS estimates are from a data assimilation product^20^. The multi-model mean from fifteen CMIP5 coupled climate models are also shown (individual model results appear in Figs SI4, SI5 and SI6). In (**b**) seasonality numbers for NSIDC and HadISST Antarctic sea ice extent are plotted. The thin lines show the seasonality numbers for NSIDC sea ice area. Notice the stretched scale for the period of satellite data.

**Figure 2:**
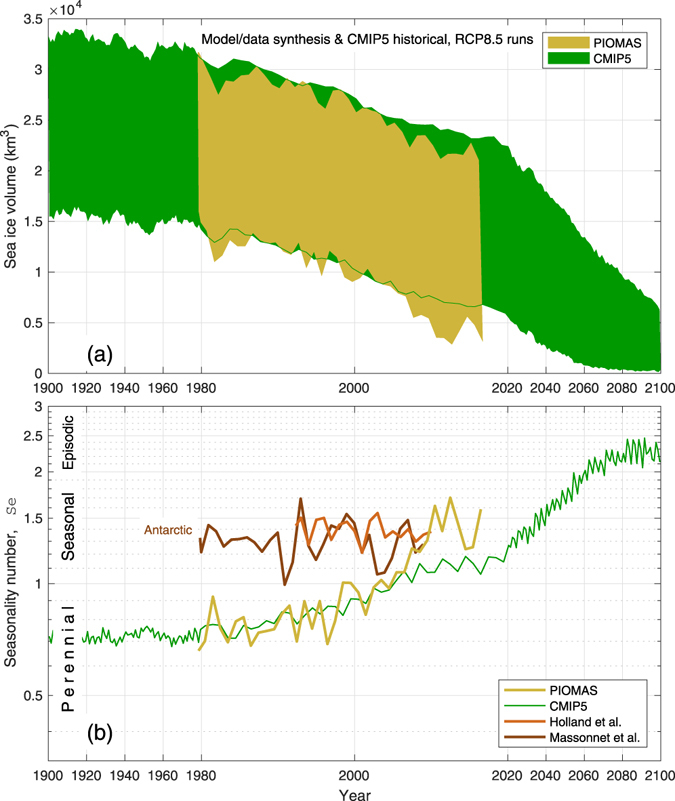
Increasing seasonality of Arctic sea ice volume (otherwise, as Fig. 1). Two sets of seasonality numbers for Antarctic sea ice volume are shown for comparison in (**b**), one derived from a sea ice-ocean model simulation^23^ (brown) and one based on a sea ice assimilation product^24^ (dark brown).

Evidence for Arctic sea ice decline comes from multiple sources, including satellite data, *in situ* observations, and coupled climate models. For example, the linear trend in northern hemisphere monthly-mean sea ice extent in summer and autumn is −6.6 ± 1.2% per decade on average (for 1979–2012^1, 2^; see also Fig. 1a). The corresponding rate of decline in winter and spring is much lower at −2.1 ± 0.5% per decade. Arctic sea-ice is also getting thinner^3, 4^. The mean thickness at the time of minimum extent has decreased from 3.02 m (during 1958–1976, from submarine data), to 1.43 m (during 2003–2007, based on ICESat satellite data). As a consequence of decreasing extent and thickness, Arctic sea ice volume is dropping too^5^, by 20% for the mean volume of the 2000s compared to 1980–2000 (based on the PIOMAS sea ice assimilation product; see also Fig. 2a).

The rate of Arctic sea ice decline projected by coupled climate models in the 21st century varies greatly^6, 7^. Many models still have difficulties to simulate a trend similar to the observed rapid ice loss^8, 9^. Nevertheless, a subset of models among those at the Coupled Model Intercomparison Project phase 5 (CMIP5) demonstrate realistic sea ice declines (Figs 1a, 2a, SI4 and SI5). Moreover, the sea ice decline has been attributed to anthropogenic CO_2_ forcing in the atmosphere, not internal variability or unforced instability due to ice-albedo feedback^10, 11^. On the basis of this evidence, the Arctic sea ice system will switch from a regime of perennial (year-round) ice cover to seasonal cover (mainly in winter) in the 21st century^6, 12–14^. For example, sea ice projections in the fourth assessment of the Intergovernmental Panel on Climate Change (IPCC) suggest that a large part of the Arctic Ocean will be seasonally ice-covered by the end of the twenty-first century^7^.

To quantify this change in seasonality, attention has focused on when the Arctic will be ice-free in summer. Typically, this question is addressed by estimating when September sea ice extent is less than 10^6^ km^2^ (about 15% of the 1980s value) for at least five consecutive years^8, 9, 15, 16^. For example, analysis of sea ice projections in the fifth IPCC assessment climate models^16^ suggests that this threshold will be reached in 2054–2058 under a high emission scenario (the representative concentration pathway 8.5 scenario, RCP8.5^17^). Under a medium-mitigation scenario (RCP4.5) September sea ice extent is projected to reach 1.7 × 10^6^ km^2^ in the early 2060s, followed by a leveling off^16^. Nevertheless, model-based estimates of when the Arctic will be ice-free in September are associated with substantial uncertainty^8, 9, 14^.

**Figure 3:**
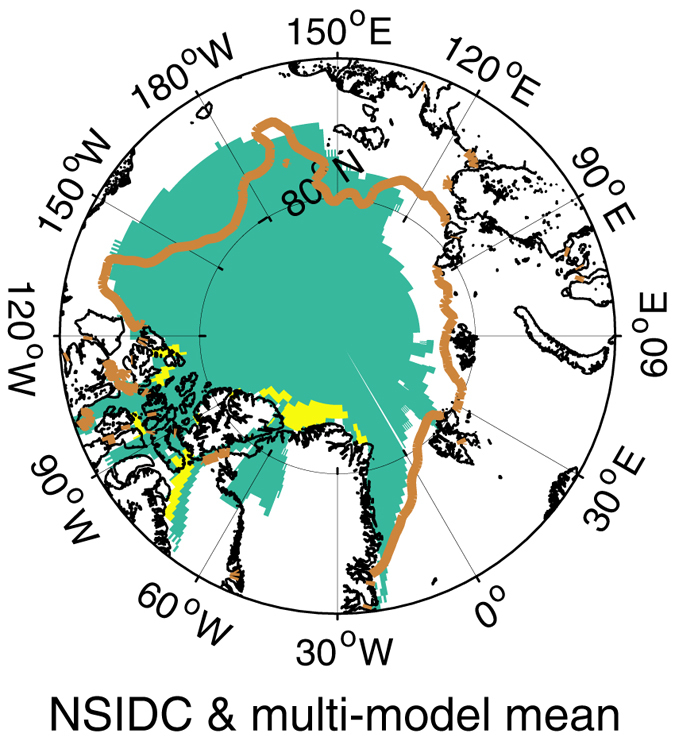
Map of sea ice extent when *Se* = 1 ± 0.05 (the brown line shows the NSIDC ice edge and the turquoise patch is from the CMIP5 models). The yellow patch shows the CMIP5 distribution of sea ice extent when the total extent decreases to (1 ± 0.6) × 10^6^ km^2^. In each case, the composite multi-model mean distribution is shown. The map was created using MATLAB R2016b software (www.mathworks.com/products/matlab.html) and the M_Map mapping package (www.eoas.ubc.ca/~rich/map.html).

Although an important issue, this threshold approach emphasizes the final stages of the transition, when the summer sea ice is about 85% gone. It does not address the earlier stages of the transition which involve dramatic changes to the Arctic climate system. Furthermore, the ice-free-summer threshold is a binary criterion that cannot measure the continuous transition in the sea ice seasonal cycle. It relies on a somewhat arbitrary (albeit reasonable, see Fig. 3) definition of “ice-free;” namely the 10^6^ km^2^ threshold on summer ice extent. The date at which the Arctic is declared to be ice free depends on this threshold in an obvious way.

In this paper we propose a novel, non-dimensional number to quantify seasonality as an objective, continuous indicator of Arctic change. We then apply it to the Arctic-Subarctic sea ice system and explore the implications for Arctic amplification of global warming. The key findings are that the Arctic-Subarctic sea ice system is now as seasonal as the Antarctic sea ice system, and we expect that surface-based autumn Arctic amplification will peak in the present decade.

## Proposed Seasonality Number

The *seasonality number*, *Se*(*T*), of a variable *x*(*t*), such as sea ice extent, is the seasonal change (the range) in *x* for a given year *T* divided by its mean value over the season,

1 $$Se(T)=({t}_{2}-{t}_{1})\frac{|x({t}_{2})-x({t}_{1})|}{{\int }_{{t}_{1}}^{{t}_{2}}\,x(t^{\prime} )\,dt^{\prime} }.$$

Here *t*_1_ and *t*_2_ are the times of consecutive extreme values of *x*, typically separated by about six months. By definition, |*x*(*t*_2_) − *x*(*t*_1_)| equals the seasonal range between the monthly-mean winter maximum sea ice extent *x*(*t*_1_) and the summer minimum *x*(*t*_2_), and ${\int }_{{t}_{1}}^{{t}_{2}}\,x(t^{\prime} )dt^{\prime} /({t}_{2}-{t}_{1})$ is the mean sea ice extent between the winter and summer extrema. The seasonality number *Se*(*T*) is plotted against time *T* for each year. Further discussion of the seasonality number, including pedagogical examples (in Fig. SI1) and a summary of its properties, appears in the Supplementary Information.

## Results

### Sea Ice Extent Seasonality

Observations and CMIP5 model simulations of the Arctic-Subarctic, i.e. the total northern hemisphere, sea ice extent and the corresponding seasonality numbers are shown in Fig. 1. *Sea ice extent* is defined as the total area enclosed by the 15% sea ice concentration contour. The observations are from the monthly National Snow and Ice Data Center (NSIDC; see Methods) satellite sea ice index^18^. They show the well-known decline in summer-time sea ice extent in recent years, from annual minima around 7 × 10^6^ km^2^ in the early 1980s to record-breaking lows of about 4.3 × 10^6^ km^2^ and 3.6 × 10^6^ km^2^ in 2007 and 2012. In contrast, the winter-time sea ice maxima losses are smaller. Prior to the satellite observations, sea ice estimates are taken from a recent product^19^ of gridded historical ice observations (see Methods), which show weaker trends.

Figure 1a also shows sea ice extent data from the Polar Science Center Pan-Arctic Ice Ocean Modeling and Assimilation System (PIOMAS^20^). The PIOMAS estimates cover the same time period as the NSIDC data, but the seasonal range is less; mainly because they underestimate the winter-time maxima in sea ice extent. The summer-time PIOMAS minima are also typically greater than from the NSIDC data, but they nearly coincide since 2007.

The associated sea ice extent seasonality numbers appear in Fig. 1b. Prior to 2000, the NSIDC data exhibit seasonality numbers around 0.75. This means that the seasonal range (winter maximum minus summer minimum) in sea ice extent was about 75% of the mean sea ice extent. The seasonality numbers from the PIOMAS estimates are also in the perennial regime (*Se* < 1; Supplementary Information), but are lower, around 0.55. Although the PIOMAS sea ice extent means are smaller than those from the NSIDC data, the NSIDC seasonal ranges are larger (Fig. 1a), which dominates the seasonality number (Eq. (1)). Since 2000, the seasonality numbers from both the NSIDC and the PIOMAS have increased, mainly because the seasonal range has increased (Fig. SI3). The NSIDC seasonality number peaked above one in 2007 and 2012, the years of record low summer sea ice extents. The PIOMAS seasonality number matches the NSIDC value much closer since 2007.

For comparison, Fig. 1b also shows the seasonality number for NSIDC sea ice extent in the Antarctic. In this case, *Se* ≈ 1.3 with no obvious trend over the last century (prior to the satellite observations, sea ice estimates are taken from the HadISST product^21^; see Methods). The Antarctic sea ice system is well-known to be in the seasonal regime^1, 22^, although other studies have not quantified the seasonality with an objective metric like *Se*. The Arctic-Subarctic sea ice extent is now approaching the same degree of seasonality, with fluctuations, and in 2012 it peaked at *Se* = 1.16, overlapping the envelope of Antarctic values. The seasonality number for NSIDC *sea ice area* (the total area covered by ice) for the northern and southern hemispheres is shown in Fig. 1b with thin brown lines. Sea ice area *Se* is very well correlated with *Se* for sea ice extent. It is greater, however, by about 0.19 on average. The reason is that the mean sea ice area is less than the mean sea ice extent in general, so the denominator in (1) is smaller for sea ice area. Still, the northern hemisphere sea ice area seasonality now overlaps the Antarctic values, as it does for sea ice extent.

Results from fifteen CMIP5 coupled climate models are also shown in Fig. 1. We use historical and RCP8.5 projections from those models that exhibit realistic Arctic sea ice area during the period of overlap with the NSIDC data considering various validation metrics^14, 16^. Specifically, these models meet criteria on realism of their September ice extent, their trend in September ice extent, their monthly climatology and the magnitude of their seasonal cycle. For the 20th century, the multi-model mean shows realistic sea ice extent cycles between 8 and 16 × 10^6^ km^2^ (results from the individual model runs are in Figs SI4 and SI6). The selected CMIP5 models all show declines in sea ice extent at the end of the 20th and throughout the 21st centuries. They project that summer minimum sea ice extent will fall below 10^6^ km^2^ by about 2070. As in the observations, the CMIP5 winter-time (March mean) declines are smaller than those in the summer-time (September mean). The rates of decline are smaller than the NSIDC data in the summers of the 2010s.

The sea ice extent seasonality number for the CMIP5 multi-model mean is around 0.7 for 1900 to 1995, then increases (Fig. 1b). The CMIP5 model seasonality number trends over the period of NSIDC data are realistic, as is the pattern of sea ice extent when *Se* passes one (Fig. 3, turquoise area). The inter-annual variability in the sea ice extent seasonality number of the CMIP5 models selected is also realistic compared to the NSIDC data (0.064 compared to 0.068). The CMIP5 multi-model mean seasonality number moves to the seasonal regime in the 2020s, however, about ten years after the NSIDC data mainly because they under-estimate the loss of sea ice extent in the 2010s.

The dominant factor in controlling *Se* for the Arctic for the observational period is the annual minimum in sea ice extent (namely, the *multi-year* *sea ice* *extent*). Indeed the linear correlation coefficient between these variables is −0.97 for the NSIDC data. The linear correlation fails for multi-year sea ice extent less than about 2 × 10^6^ km^2^ (for the CMIP5 models), however, and for the Antarctic data in Fig. 1. The Supplementary Information and Fig. SI2 provide details.

### Sea Ice Volume Seasonality

Corresponding sea ice volume estimates from PIOMAS and the CMIP5 models are in Figs 2 and SI5. PIOMAS sea ice volume shows declines in both winter- and summer-time extremes through the whole record. The rate of decline exceeds that of sea ice extent (Fig. 1) because sea ice volume depends on the product of sea ice area and thickness, which are both declining. The PIOMAS seasonality number for sea ice volume (Fig. 2b) shows a rapid increase from about 0.75 in the 1980s to about 1.5 by the end of the record, mainly because the seasonal mean has decreased (Fig. SI3). Two recent model estimates of Antarctic sea ice volume seasonality numbers appear in Fig. 2b; from a forced ice-ocean simulation^23^ and using assimilation^24^, essentially similar to the PIOMAS. For most of the last decade, the PIOMAS seasonality numbers have been in the same range as the Antarctic estimates. The PIOMAS Arctic-Subarctic sea ice volume seasonality exceeds the highest Antarctic *Se* in 2012, however. The CMIP5 multi-model mean sea ice volume seasonality numbers agree well with the PIOMAS estimates although they under-estimate the rate of increase in the 2010s. The RCP8.5 sea ice volume projections enter the seasonally-episodic regime (*Se* > 2; Supplementary Information) after about 2060.

### Implications for Arctic Amplification

**Figure 4:**
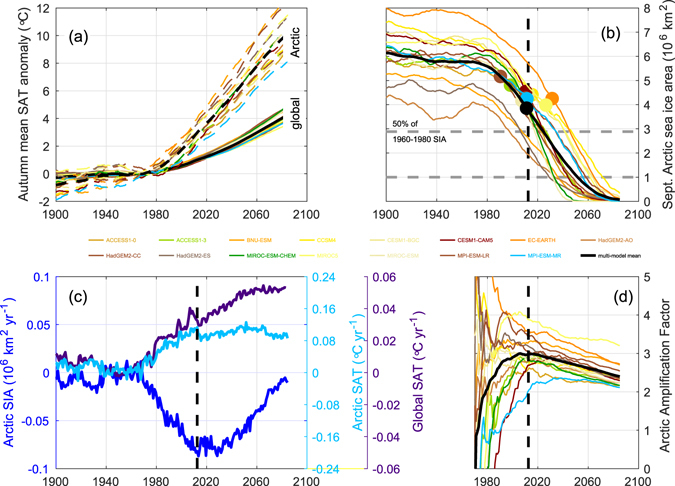
Sea ice trends and peak Arctic amplification in CMIP5 historical runs and RCP8.5 projections. (**a**) Autumn (September to November) Arctic (north of 70°N) mean surface air temperature (SAT; dashed lines) and global mean SAT (solid lines). Temperatures are anomalies with respect to the relevant 1960–1980 mean and smoothed by a 30-year boxcar filter. (**b**) Arctic September mean sea ice area (SIA) from the CMIP5 models (colored lines) and the multi-model mean (black line). Colored circles mark the year in which each model run passes through *Se* = 1. The vertical black dashed line marks the multi-model mean peak year of sea-ice-related feedbacks (also shown in (**c**,**d**); see Fig. SI7). The lower horizontal gray dashed line marks the commonly-used 10^6^ km^2^ threshold of an ice-free Arctic. The upper horizontal gray dashed line marks 50% of the 1960–1980 SIA value. (**c**) Rates of SIA and SAT change based on the 30-year running mean smoothed multi-model mean time series shown in (**a**, **b**). (**d**) Time series of the Arctic amplification factor defined as the ratio of Arctic to global SAT anomalies as shown in (**a**).

Seasonality in the Arctic-Subarctic sea ice system affects diverse physical, chemical, and biological processes such as surface ocean warming^25, 26^ with implications for sea ice melt^27^, atmospheric moisture and cloud cover^28, 29^, momentum transfer into the ocean^30, 31^, primary production^32^, and ecological dynamics^33^. We illustrate the impact of the transitioning of the Arctic sea ice cover to the seasonal regime on the climate system by a simple example: the role of the ice cover in Arctic amplification. Arctic amplification is the faster rate of Arctic warming compared to the global average (Fig. 4a), which is most prominent in autumn and winter^34, 35^. While Arctic amplification can be found throughout the troposphere, it is strongest at the surface^35^. Several studies show that sea ice plays a major role in surface Arctic amplification^36–39^. Here, we focus on the relationship of the September-mean sea ice area and surface air temperature (SAT) during autumn (September to November mean).

Sea ice influences, among other variables, Arctic SAT by moderating both absorption of insolation at the surface and property exchange between the ocean and atmosphere. As sea ice retreats during the melt season it exposes the darker ocean to direct insolation, which then warms locally over the summer, causing further ice retreat. Seasonally, the warmer ocean accelerates ice melt and delays re-freezing. Inter-annually, less sea ice survives summer melt and the shorter freezing season promotes overall thinner ice which is more prone to retreat during the next melt season. This positive ice-albedo feedback is an important process under global warming^34, 40, 41^.

The sea ice cover also impedes heat and moisture fluxes from the ocean to the atmosphere. In autumn and winter the open ocean can significantly warm the atmosphere in the absence of sea ice^42^. In conjunction with advection from lower latitudes of warm and moist air, this was a key factor leading to unusual high surface temperatures in the Arctic in autumn 2016^43^. Moreover, it shows that Arctic amplification is influenced by non-local processes. Sea ice retreat, and its consequences, may cause it to vary in time, however. For example, the positive ice-albedo feedback is most effective when a thin ice cover exists because it requires the contrast between dark open water that warms and bright sea ice that rapidly melts. Due to the topographic enclosure of the Arctic Ocean by land, sea ice retreat is limited and the ice-albedo feedback will disappear once the ocean becomes ice-free in the sunlit season. To the extent that the ice-albedo feedback influences Arctic amplification, Arctic amplification should be greatest during the time of fastest year-to-year ice retreat.

To investigate this idea further we focus on two quantitative diagnostics. We study the decadal increase in autumn mean surface air temperature (SAT) north of 70°N (Fig. 4a, dashed lines) and the decline in summer minimum sea ice area (SIA; Fig. 4b). By limiting the area of interest to north of 70°N we focus on processes related to the Arctic Ocean, such as sea ice coverage and enhanced upper ocean heat storage. Other processes involved in Arctic amplification, such as variations in snow cover on land, are not explicitly considered, although they are represented in the climate models. The CMIP5 model RCP8.5 projections are examined after applying a 30-year boxcar filter to reduce the influence of model-specific interannual to decadal variability. The rates of change of both Arctic summer SIA and autumn SAT peak in the decade of the 2010s (Fig. 4c, blue and light blue lines). In contrast, the rate of change of global mean autumn SAT increases to a plateau after 2060 (Fig. 4c, indigo line).

We quantify the peak effectiveness of sea ice feedbacks using the year of greatest combined summer sea ice retreat and subsequent autumn warming (see Methods and Fig. SI7). This peak occurs in 2012 for the CMIP5 multi-model mean and is shown with a black vertical dashed line in Fig. 4b–d. At the same time autumn Arctic SAT warming reaches a maximum and so does Arctic amplification, measured by an Arctic amplification factor, which reaches a magnitude of 3.0 in 2015 for the ensemble mean (Fig. 4d). We define the Arctic amplification factor as the ratio of Arctic mean to global mean SAT anomaly. This variable varies with time to quantify the secular change in the amplification magnitude. SAT anomalies are computed with respect to the period 1960–1980. Autumn Arctic SIA decline stays high for another ten years before diminishing after 2030.

Importantly, the peak year in sea ice feedback effectiveness coincides with the year that the CMIP5 multi-model mean passes from the perennial to the seasonal regime for sea ice area (*Se* = 1, coloured circles on Fig. 4b). And it precedes both the time when half the September sea ice area is lost and when the commonly-used 10^6^ km^2^ threshold is reached (in years 2024 and 2050, respectively, for the CMIP5 multi-model mean). This finding suggests that the seasonality number is closely related to the effectiveness of sea ice feedbacks in modulating Arctic amplification. The multi-model mean Arctic amplification factor decreases significantly after 2015 reaching a value of 2.4 toward the end of the study period in 2085 (Fig. 4d).

Processes such as the ice-albedo feedback preferentially accelerate summer sea ice melt, increasing the seasonal range, namely, the numerator in (1). They also facilitate an inter-annual decrease of the mean ice thickness and area, thus decreasing the denominator of (1). Both effects increase the seasonality number, with greatest impact when sea ice processes are most effective, i.e. when there is still a contrast between ice-covered and open ocean in the Arctic. Thus it is reasonable to hypothesize that the change from the perennial to the seasonal regime occurs when sea ice feedbacks are most effective. For these reasons, we speculate that the decisive transition of the Arctic climate and eco-systems will occur during the next decade, long before the threshold of an ice-free Arctic is reached.

## Discussion

We attribute the reduction of autumn Arctic surface warming and the reduction in surface-based Arctic amplification to summer sea ice loss. The processes involved include, among others, a reduced ice-albedo feedback and reduced capability of sea ice to moderate ocean-atmosphere exchange. This is a plausible explanation because the timing of peaks in Arctic SAT growth rate, Arctic SIA loss rate, and Arctic amplification coincide (Fig. 4c,d). The ice-albedo feedback (as part of the surface-albedo feedback) has been cited as a principal reason for Arctic amplification of anthropogenic climate change^35, 41, 44^. Nevertheless, it coexists with other (partly-dependent) feedback mechanisms known to cause Arctic amplification. They include sea ice processes unrelated to albedo (involving modified sea surface temperature), the atmospheric lapse rate feedback^45^, temperature effects^46^, and surface albedo change due to adaption processes in clouds, snow cover and vegetation^34^.

Future research should investigate the causal link between the loss of summer Arctic sea ice, concomitant albedo decrease, ocean heat uptake, ocean surface warming, and long-wave radiation, and the reduction in Arctic amplification seen in Fig. 4. For example, recent work^47^ describes the link between sea ice loss and surface ocean warming, emphasizing the importance of the timing of ice retreat. In the 2010s ice retreat occurs earlier in the marginal shelf seas of the Arctic compared to the 1980s, which enables greater warming there. In the central Arctic Ocean, ice retreat commences late in summer, after August 1, when the net heat flux into the ocean is weaker and increasing winds stir the ocean more strongly. For a significant impact on Arctic amplification, these regions need to become ice-free at least six weeks earlier to substantially warm over the summer^47^. Since the Arctic has already transitioned into a state of predominantly first-year ice^48^ we hypothesize that this timing sensitivity is most effective in the 2010s, adding to the current peak in Arctic amplification.

The CMIP5 models considered here exhibit realistic sea ice properties compared to the NSIDC data^14, 16^. We select these models because our focus is on the presently-occurring transition from the perennial to the seasonal ice regime, and the associated peak in Arctic amplification. Clearly, uncertainty grows projecting into the future. Indeed, the set of CMIP5 models selected here may not have the most realistic projections^49^.

Regardless, the real Arctic sea ice system passed into the seasonal regime recently (Figs 1 and 2), as summer ice diminishes. The Arctic sea ice feedback mechanisms are therefore also decreasing. The CMIP5 models that capture such changes in Arctic sea ice exhibit an Arctic amplification history smoothed over 30 years that reaches a maximum in 2012. The model Arctic amplification then declines over a few decades as the sea ice feedback mechanisms fade away. On this basis, it is reasonable to suppose that the amplification of global warming in the real Arctic is also peaking.

## Methods

### Sea Ice Data

The NSIDC data product comprises merged satellite passive microwave measurements from the Defense Meteorological Satellite Program (DMSP) Special Sensor Microwave Imager/Sounder, the older DMSP Special Sensor Microwave/Imager, and the Nimbus-7 Scanning Multichannel Microwave Radiometer. The NASA Team algorithm is used to derive sea ice concentrations. These data (version 2.1 for the monthly sea ice index data, version 1.1 for the daily data) are available since November 1978. Prior to that time, we use the Walsh *et al*.^19^ gridded monthly sea ice data which synthesizes the (sparse) records of sea ice extent, primarily from hand-drawn charts^50^. Similarly, we use HadISST data^21^ for the Antarctic before the satellite era. The PIOMAS product^20^ blends ice concentration and sea-surface temperature data with a dynamical sea ice and ocean circulation model.

Sea ice extent is defined as the area of ocean enclosed by the 15% sea ice concentration contour, which is computed from the sea ice distribution. Sea ice area is the integrated area covered by ice. It is a metric that avoids the non-linearity inherent in sea ice extent, but has a larger observational uncertainty. The correlation coefficient for northern hemisphere sea ice extent and sea ice area is 0.94. Sea ice volume is the area-integrated product of sea ice concentration (ice-covered area fraction) and thickness and is harder to estimate than either sea ice extent or area because of the uncertainty in ice thickness.

The NSIDC satellite data contain various uncertainties, but they all cause small errors compared to the inter-annual changes and the decadal trend seen in the seasonality number in Fig. 1b. First, a region near 90°N is unobserved by the satellites (the so-called “pole-hole”). Various assumptions exist to extrapolate the sea ice concentration over the unobserved region, but they do not affect the seasonality number. Second, uncertainty in sea ice concentration for individual grid cells in the daily sea ice images (between 5 and 15%, depending on season) causes uncertainty in the seasonality number that is smaller than the thickness of the line in Fig. 1b. Consistently, the uncertainty on total sea ice extent is 2–3 × 10^4^ km^2^ (W. Meier, Personal Communication), which gives an uncertainty in *Se* of a few parts in a thousand. Finally, different algorithms exist to process the satellite data, but they do not cause large uncertainty in the seasonality number: for example, the NASA Bootstrap sea ice product (version 2.1)^51^ gives an *Se* timeseries that differs from that of the NSIDC data in Fig. 1b by 0.028 (smaller) on average, a standard deviation of 0.016, and a correlation coefficient of 0.993.

### Peak Sea Ice Impact on Surface Air Temperature

Year-to-year differences of summer minimum (here, September mean) sea ice area (SIA) and autumn (September through November mean) surface air temperature (SAT) anomalies (with respect to the 1960–1980 mean) averaged over the Arctic Ocean (here, the area north of 70°N) and globally are computed from CMIP5 model output which is smoothed with a 30-year boxcar filter. Year-to-year differences are displayed in Fig. 4c as time series and in Fig. SI7 as scatter plots. The scatter diagram of Arctic SIA versus SAT is used to compute a normalized distance *d* from the origin for each year *T*, as follows (Fig. SI7):

2 $$d{(T)}^{2}={[\frac{{\rm{\Delta }}\mathrm{SAT}}{{\rm{\max }}(|{\rm{\Delta }}\mathrm{SAT}|)}]}^{2}+{[\frac{{\rm{\Delta }}\mathrm{SIA}}{{\rm{\max }}(|{\rm{\Delta }}\mathrm{SIA}|)}]}^{2},$$

with

3 $${\rm{\Delta }}\mathrm{SAT}={{\rm{SAT}}}_{T}-{{\rm{SAT}}}_{T-1}\,{\rm{and}}\,{\rm{\Delta }}\mathrm{SIA}={{\rm{SIA}}}_{T}-{{\rm{SIA}}}_{T-1}.$$

The algorithm is applied to the CMIP5 model realizations combining the historical runs and RCP8.5 climate projections and to the multi-model mean (black vertical dashed lines in Fig. 4b–d; black × in Fig. SI7).

We use a 30-year running mean to low-pass filter the CMIP5 sea-ice area and surface air temperature records. The intention is to focus on the trend forced by the increase of greenhouse gases (RCP8.5 scenario) and to reduce the impact of interannual to decadal internal variability. This procedure does not fully eliminate internal variability, however, which differs between models^52^.

## Electronic supplementary material

Supplementary information pdf
