# Supplementary material for: The Arctic-Subarctic sea ice system is entering a seasonal regime: Implications for future Arctic amplification
Source: Sci Rep. 2017 Jul 4;7:4618. doi: 10.1038/s41598-017-04573-0 (PMC5496918; doi:10.1038/s41598-017-04573-0)
Supplement: Supplementary file 1 — Supplementary information pdf [file 41598_2017_4573_MOESM1_ESM.pdf]

# Supplementary Information for: The Arctic-Subarctic sea ice system is entering a seasonal regime: Implications for future Arctic amplification

**Thomas W. N. Haine**[1,*] **and Torge Martin**[2]

[1]Earth & Planetary Sciences, The Johns Hopkins University, Baltimore, MD USA
[2]GEOMAR Helmholtz Centre for Ocean Research Kiel, Kiel, Germany
[*]Thomas.Haine@jhu.edu
[+]These authors contributed equally to this work.

## ABSTRACT

Supplementary information on the seasonality number and supporting figures.

## Discussion of Seasonality Number

### *Definition*

The *seasonality number*, $Se(T)$, is defined by equation (1) of the main text. We consider a non-negative, time-dependent variable $x(t)$ which is (quasi-)periodic, for example with a period of 12 months. The seasonality number is the ratio of two quantities, the seasonal range $\Delta x$ in the variable for a given year $T$,

$$\Delta x = x(t_2) - x(t_1), \tag{SI1}$$

and the average value over the season, $\bar{x}$,

$$\bar{x} = \frac{1}{t_2 - t_1} \int_{t_1}^{t_2} x(t')\, dt'. \tag{SI2}$$

Namely,

$$Se(T) = \frac{|\Delta x|}{\bar{x}}. \tag{SI3}$$

In these formulae $t_1$ and $t_2$ are the times of consecutive extreme values of $x$, typically separated by about six months. We take the absolute value in the numerator of (SI3) to suppress the negative sign of $Se$ for cases of seasonal decrease in $x$ (when $x(t_2) < x(t_1)$, which we apply throughout). The seasonality number is a function of $T$ because it can vary between successive pairs of extrema in $x(t)$.

### *Pedagogical Examples*

Consider the simple examples in Figure SI1 to make the seasonality number concept clear. The upper two panels show annual periodic cycles with maxima and minima of $x(t_1) = 16$ and $x(t_2) = 7$, and hence $\Delta x = -9$. They are inspired by the observed 1980s–1990s winter Arctic sea ice extent ($16 \times 10^6$ km$^2$) being about twice the summer extent ($7 \times 10^6$ km$^2$). The upper panel shows sinusoidal and sawtooth periodic functions. Although the times of their maxima and minima differ, they have identical ranges and mean values ($\bar{x} = 11$), so their seasonality numbers are the same and equal 0.78. These cases demonstrate the point that $Se$ is independent of the shape of the periodic function $x(t)$, so long as the range $\Delta x$ and the mean $\bar{x}$ are unaffected. The middle panel shows a clipped sinusoidal function, also with $x(t_1) = 16$, $x(t_2) = 7$, and $\Delta x = -9$ (upper curve). This function accounts better for the different duration of the winter and summer seasons although still in a simplistic manner. The mean value $\bar{x} = 13$ is larger than for the sinusoidal and sawtooth functions because the distribution of $x$ values over the year for the clipped sinusoid is biased towards higher values. Therefore, the seasonality number $Se = 0.71$ is smaller than for the upper panel. The middle panel also shows a clipped sinusoid function, but with $x(t_1) = 15, x(t_2) = 4$, hence $\Delta x = -11$, and $\bar{x} = 11$,

crudely resembling the Arctic sea ice extent cycle in recent years that features a stronger decline in summer than in winter. This case has $Se = 1$. The bottom panel shows a clipped sinusoid function that might resemble the Arctic sea ice extent cycle in the coming decades. In this case, $x(t_1) = 10, x(t_2) = 0$, hence $\Delta x = -10$, and $\bar{x} = 4.8$, yielding $Se = 2.1$. The distribution of $x$ values over the year is biased low compared to a sinusoid with the same range. Therefore the mean value is smaller than $\bar{x} = 5$, which is the case for a sinusoid with the same range. Hence, the seasonality number is larger than for a sinusoid with the same range.

#### *Properties of the Seasonality Number*

These examples show that the seasonality number $Se$ is mainly controlled by the range $\Delta x$, and especially by the minimum value, not the mean value $\bar{x}$. Specifically, the middle panel of Fig. SI1 shows an increase of 2 in $\Delta x$ (lower curve) and $\bar{x}$ (upper curve), compared to the upper panel. The $\Delta x$ change impacts $Se$ three times more than the $\bar{x}$ change (a 28% increase versus a 9% decrease). The following arguments make this idea clear.

In many cases, the periodic function is evenly distributed between maxima and minima, meaning that the distribution of $x(t)$ between $x(t_1)$ and $x(t_2)$ is unbiased. Then, $\bar{x} = [x(t_2) + x(t_1)]/2$, and the seasonality number for this unbiased seasonal cycle is

$$Se_0(T) = 2\left[\frac{|x(t_2) - x(t_1)|}{x(t_2) + x(t_1)}\right] = 2\left(\frac{|\varepsilon - 1|}{\varepsilon + 1}\right), \tag{SI4}$$

where $\varepsilon = x(t_2)/x(t_1)$. For the examples in Figure SI1, $\varepsilon = 7/16 = 0.438$ (upper panel, upper curve on middle panel), $4/15 = 0.267$ (lower curve on middle panel), and $0/10 = 0$ (lower panel). Therefore, an unbiased seasonal cycle gives $Se_0 = 0.783$, as seen for the sinusoid and sawtooth functions in the upper panel. The upper clipped sinusoid in the middle panel exhibits a bias that drives $\bar{x}$ high and therefore $Se = 0.71$ is smaller than the value of $Se_0 = 0.783$ for the unbiased cycle, but still quite close. The lower clipped sinusoid in the middle panel is similar with $Se_0 = 1.16$ but $Se = 1$. In the lower panel, the unbiased seasonal cycle gives $Se_0 = 2$, somewhat less than the actual value of $Se = 2.1$ because of the low bias in $x(t)$.

This result shows that $Se > 2$ only occurs when there is a bias in the distribution of $x(t)$ so that the mean value $\bar{x}$ is smaller than $[x(t_2) + x(t_1)]/2$. In other words $Se_0 \leq 2$, which is seen in (SI4) because $|\varepsilon - 1|/(\varepsilon + 1) \leq 1$. For this reason, we refer to seasonality numbers greater than two as being in the *episodic* regime (such as Fig. SI1 lower panel), meaning that $x(t)$ only intermittently departs from near zero. There is no corresponding restriction on the smallness of $Se$ in the perennial regime $Se < 1$: as the range $\Delta x$ decreases for any minimum $x(t)$ value greater than zero, $Se$ also decreases towards zero without limit.

Now consider that the mean value $\bar{x}$ must lie between the maximum and minimum values of $x(t)$; $x(t_2) \leq \bar{x} \leq x(t_1)$ (for $\varepsilon < 1$, otherwise the inequalities are flipped). Therefore,

$$|1 - \varepsilon| \leq Se(T) \leq \left|\frac{1}{\varepsilon} - 1\right| \tag{SI5}$$

(for $\varepsilon < 1$, otherwise the inequalities flip). For the examples in the upper panel and upper curve in the middle panel of Figure SI1, $\varepsilon = 7/16 = 0.438$ and therefore $0.563 \leq Se \leq 1.29$, which is a moderately tight restriction on the seasonality number, regardless of the shape of the seasonal cycle $x(t)$. For the lower panel, which shows an $x(t)$ with a minimum value of zero, the seasonality number must satisfy $Se \geq 1$, meaning any $x(t)$ with a minimum of zero cannot be in the perennial regime, regardless of the shape of its seasonal cycle. This is a reasonable property of $Se$.

Notice that other putative definitions of the seasonality number, for example with the minimum value of $x$ in the denominator, not the average, have less desirable properties. In particular, if the denominator vanishes, as it does for Arctic sea ice in some of the CMIP5 models if the denominator is the minimum value, then the seasonality number diverges to infinity. That is less useful than the seasonality number defined by equation (1) of the main text, which naturally distinguishes between perennial, seasonal, and episodic regimes.

#### *Relation between Sea Ice Extent $Se$ and Multi-Year Ice Extent*

Figure SI2 plots multi-year sea ice extent $MYI$ against seasonality number $Se$ for the data shown in Fig. 1. Multi-year sea ice extent is defined as the minimum extent for a given year. As expected from the discussion above, there is a compact relationship between $MYI$ and $Se$. The linear correlation coefficient for the northern hemisphere NSIDC data is -0.97, which shows that over the observational record $Se$ is strongly controlled by $MYI$ (the line on Fig. SI2 is the best-fit to these data). The southern hemisphere NSIDC data are offset to higher $MYI$ values (above the line). The same is true for the southern hemisphere HadISST data, although the scatter to large $MYI$ at $Se \approx 1.25$ is presumably due to data uncertainty before the satellite era. The CMIP5 models depart from the linear correlation at $Se \gtrsim 1.3$, $MYI \lesssim 2 \times 10^6$ km$^2$. At large seasonality number ($Se \gtrsim 1.7$ , $MYI \lesssim 1 \times 10^6$ km$^2$), the multi-year ice extent no longer varies with $Se$ because it then depends mainly on the average extent (the denominator in equation SI3). Therefore, $MYI$ is a good proxy for $Se$ for the Arctic over the observational record, and until the summer minimum sea ice extent drops below about $2 \times 10^6$ km$^2$.

### *Practical Considerations*

A few practical issues should be considered when computing the seasonality number from a periodic time series. First, a clear idea of the period of interest $t_2 - t_1$ is required in order to avoid spurious extrema in $x$ at shorter periods. For annually-repeating variables, we expect $t_2 - t_1$ to be close to six months, for example. Second, using the extreme values to define the range $\Delta x$ of $x(t)$ can be problematic when the time series contains significant random noise. Then, the extrema may be sensitive to the noise in undesirable ways. Although, this problem is unlikely to severely impact the computation of the seasonality number, it may be preferable to define $\Delta x$ as the difference between (for example) the 95th and 5th percentiles of the distribution of $x(t)$ values between successive extrema. Third, for an annually-repeating variable one has the choice of defining the seasonality number each year based on the increase in $x(t)$ ($\varepsilon > 1$) or on the decrease in $x(t)$ ($\varepsilon < 1$). These two seasonality numbers may differ somewhat if $x(t)$ is biased differently for the increasing phase of its cycle compared to the decreasing phase.

### *Application to other Variables*

The seasonality number is particularly useful for sea ice and can be applied to other Earth system quantities, such as insolation, ice mass, or heat content of the upper ocean. These are extensive (additive) quantities, which are non-negative by definition. The seasonality number can be applied to intensive quantities too, but care is required. Specifically, it is not applicable to variables with an arbitrary offset. Temperature is a good example: it makes sense to talk of the seasonality number of absolute temperature measured in Kelvin (which is non-negative with a non-arbitrary zero), but not of temperature measured using an empirical scale such as Celsius or Fahrenheit.

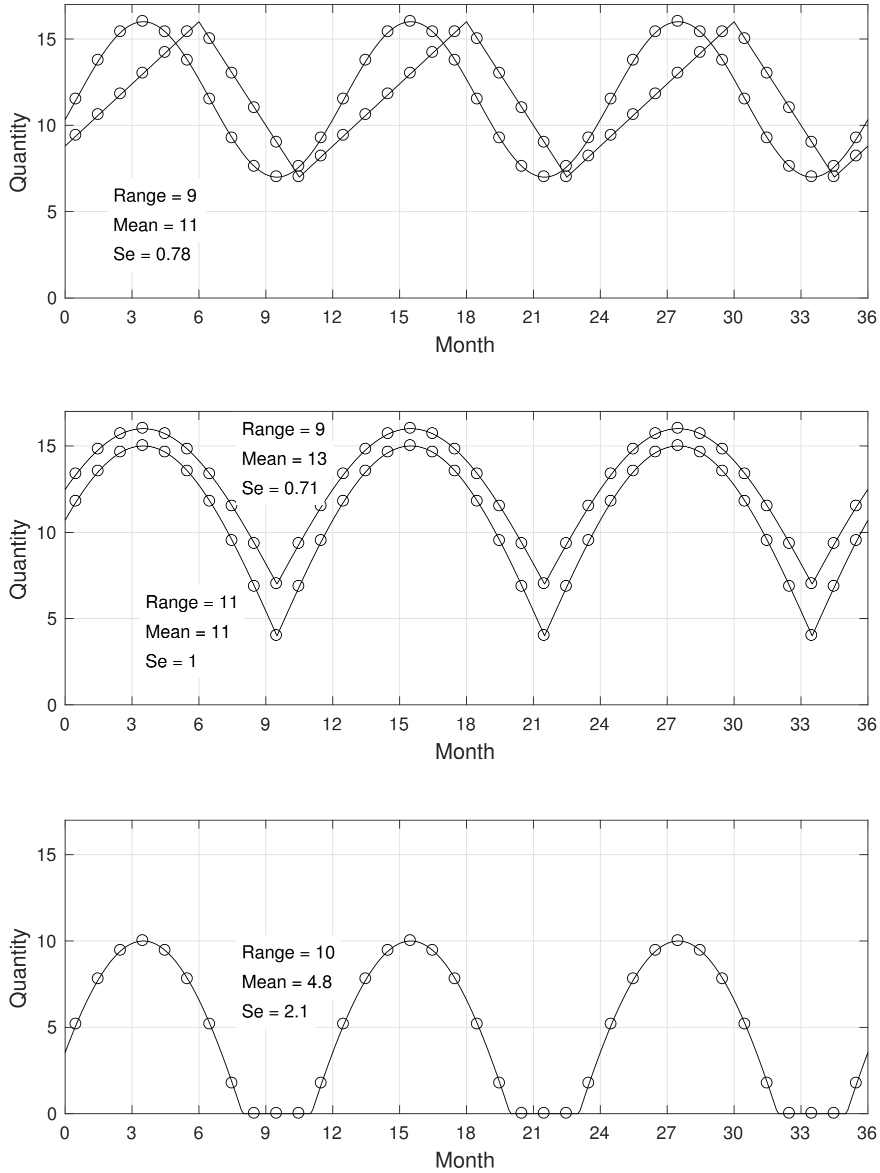


**Figure SI1.** Example annual periodic functions $x(t)$ and their seasonality numbers *Se* to illustrate the seasonality of Arctic sea ice extent. The range and mean are defined by equations (SI1) and (SI2), respectively, and *Se* is computed using (SI3).

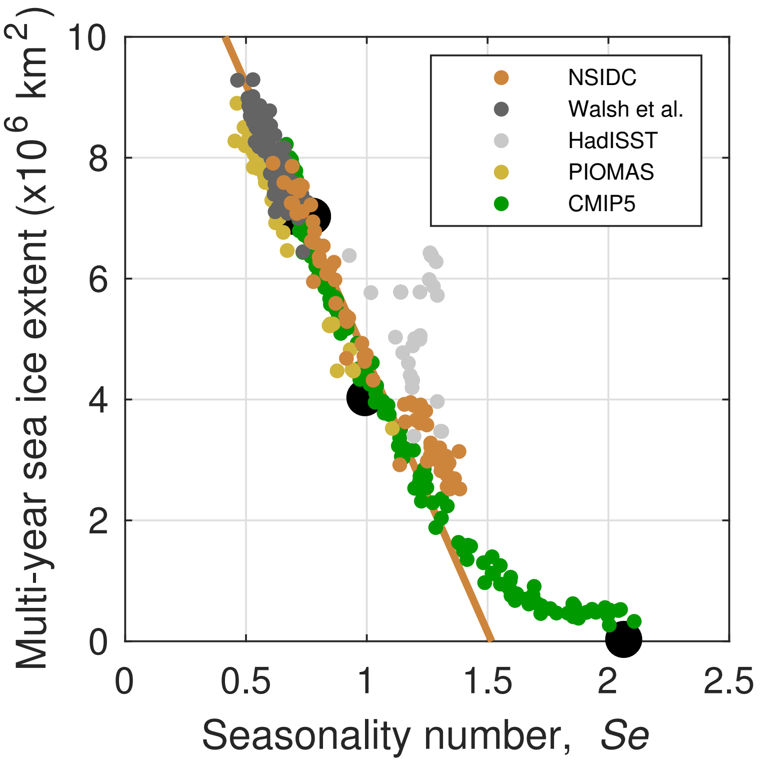


**Figure SI2.** Multi-year sea ice extent plotted against seasonality number for the datasets shown in Fig. 1. The large black dots are the pedagogical examples from Fig. SI1. The line is the best fit to the northern hemisphere NSIDC data.

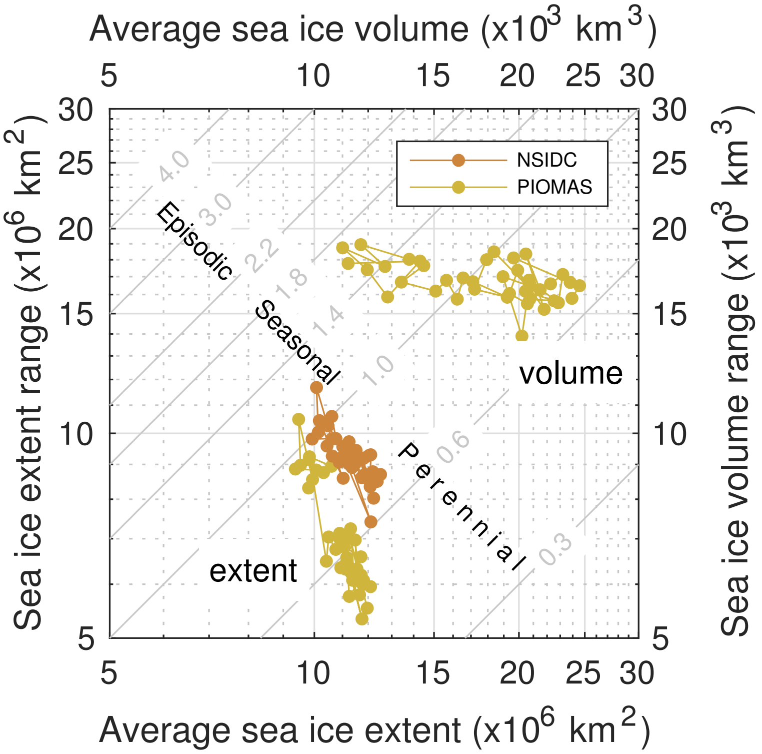


**Figure SI3.** Northern hemisphere seasonal sea ice extent (from NSIDC and PIOMAS) and volume (from PIOMAS) ranges plotted against the corresponding seasonal averages. The seasonality numbers are shown with contours. The sea ice extent and volume seasonality numbers increase with time (Figs. 1b and 2b), mainly because the ranges increase and the averages decrease, respectively.

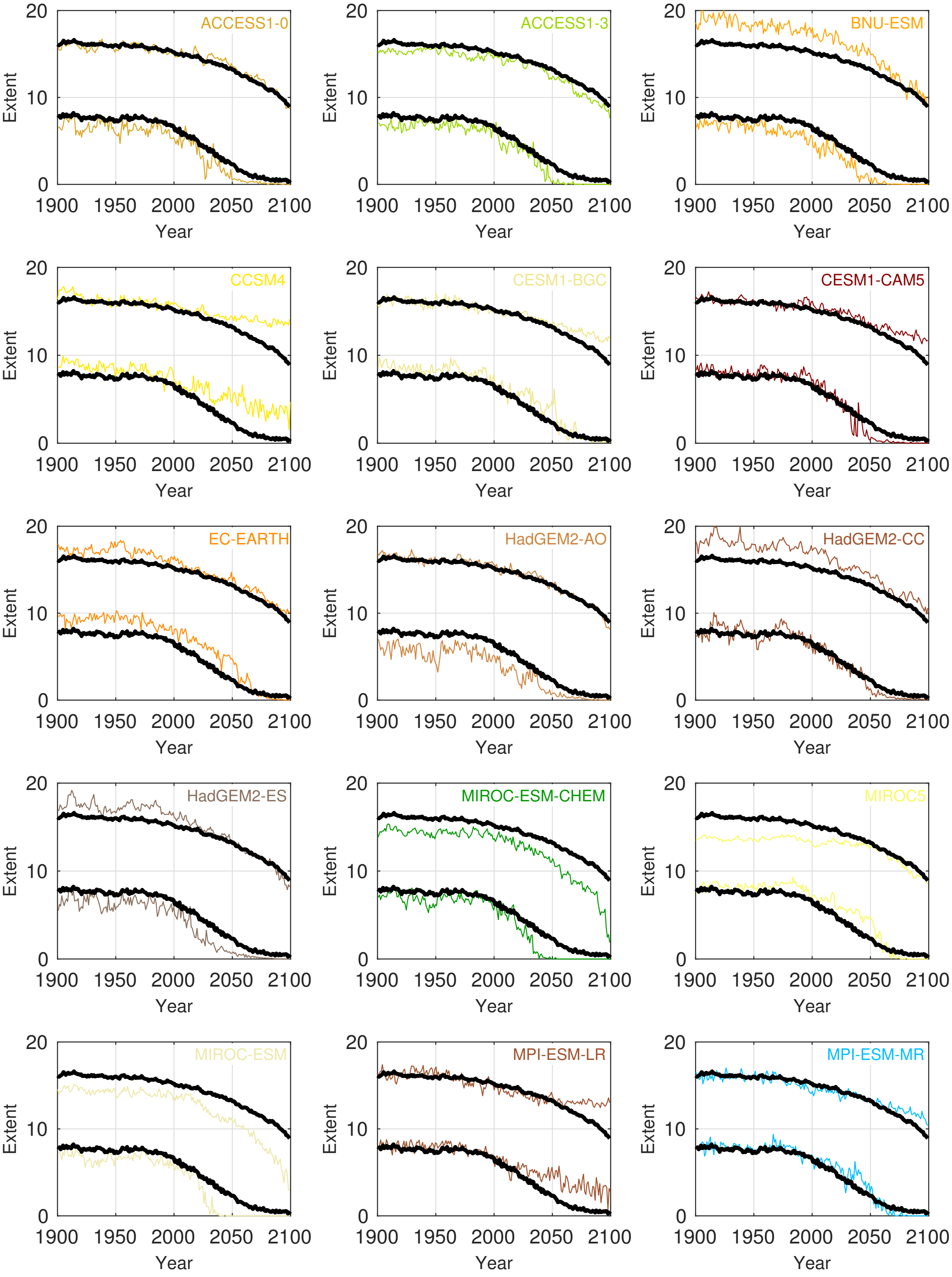


**Figure SI4.** Annual maxima and minima of sea ice extent for the CMIP5 models. The historical and RCP8.5 projections are shown. Black lines indicate the multi-model means.

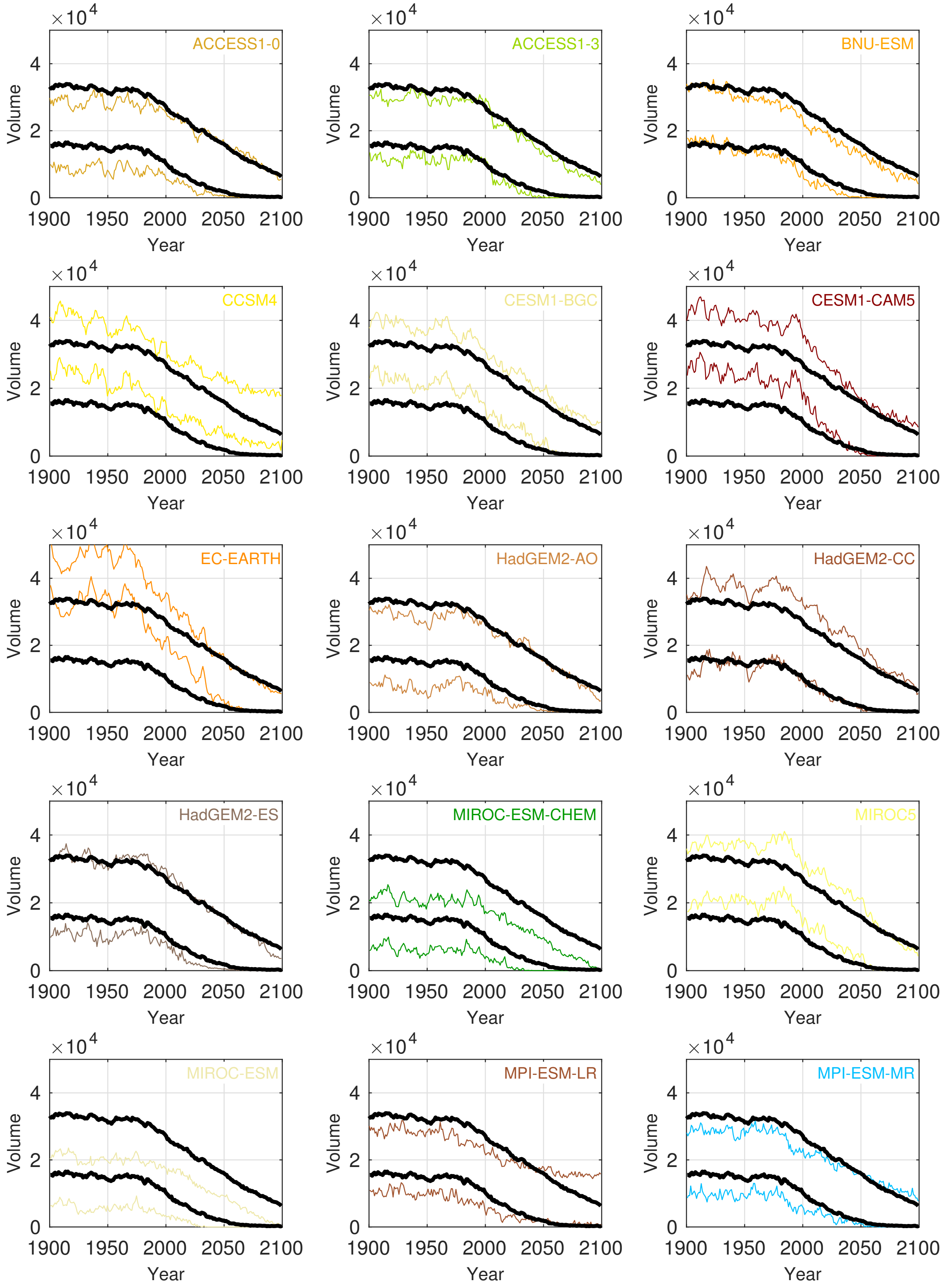


**Figure SI5.** As Fig. SI4 except for sea ice volume.

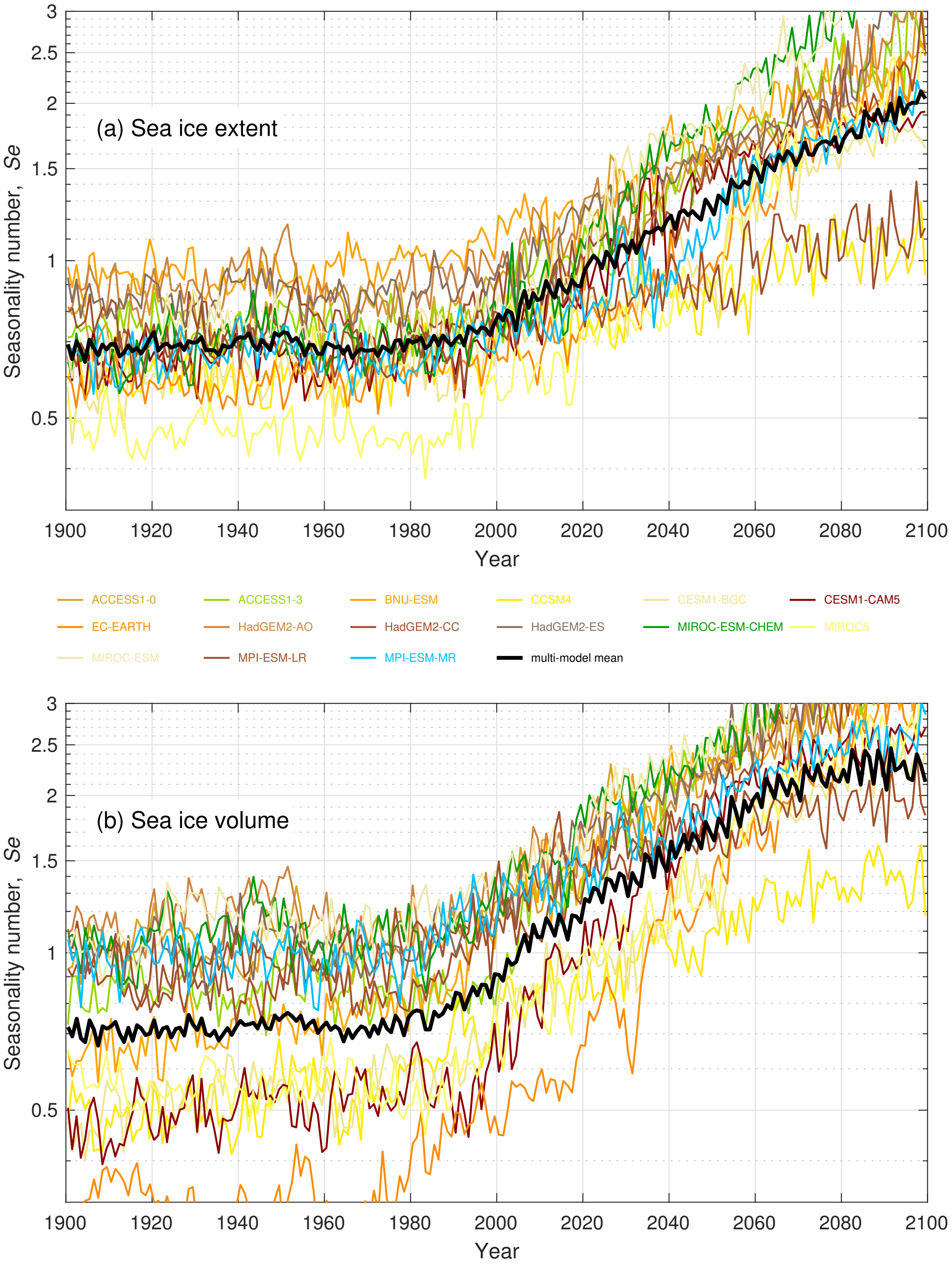


**Figure SI6.** Seasonality of Arctic sea ice extent (upper) and volume (lower) for the CMIP5 models. The black line marks the multi-model mean.

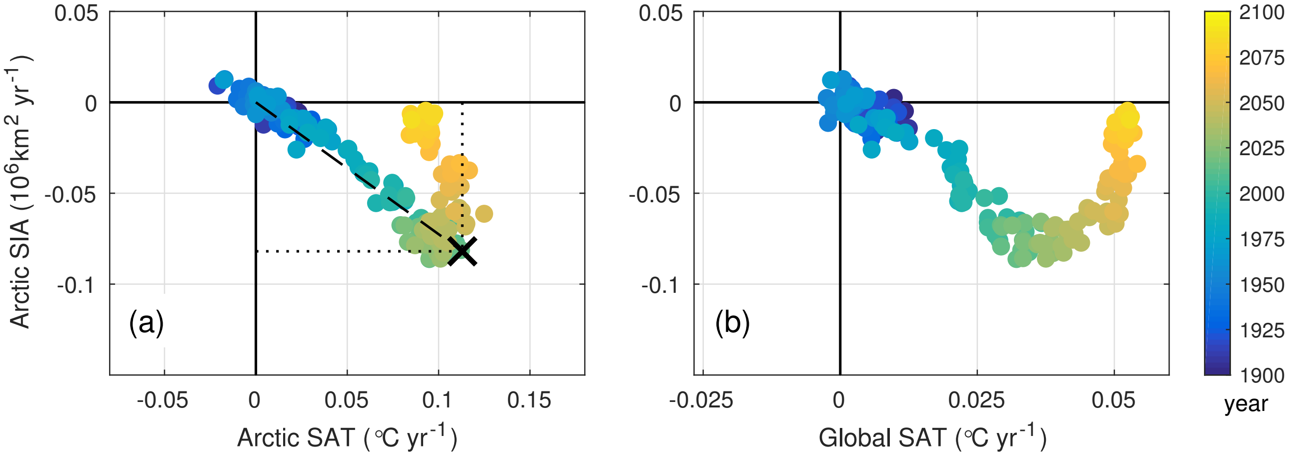


**Figure SI7.** Calculation of the peak in coincident sea-ice retreat and surface air temperature (SAT) warming. (a) Annual rate of change in Arctic (> 70°N) sea ice area (SIA) minimum as a function of the Arctic autumn (September to November) mean SAT annual rate of change (see Fig. 4c). The black × marks the year with the largest normalized distance from the origin, namely, the largest change in both SIA and SAT, which we define as the peak in the sea ice feedback (see equation (2)). The SIA and SAT data are from the multi-model mean of the CMIP5 models. (b) Same as (a) but for global autumn mean SAT (notice the different abscissa scale).
